# Editorial Perspective: Why is there such a mismatch between traditional heritability estimates and molecular genetic findings for behavioural traits?

**DOI:** 10.1111/jcpp.12294

**Published:** 2014-08-01

**Authors:** Anita Thapar, Gordon Harold

**Affiliations:** 1 MRC Centre for Neuropsychiatric Genetics and Genomics and Child &, Adolescent Psychiatry Section, Institute of Psychological Medicine and Clinical Neuroscience, Cardiff UniversityCardiff, UK; 2 Andrew and Virginia Rudd Centre for Adoption Research and Practice, School of Psychology, University of SussexFalmer, UK

**Keywords:** Molecular genetic studies, behaviour genetic studies, psychiatric disorders, behavioural traits, heritability, developmental psychopathology

## Abstract

The puzzle of the disparity between molecular- and traditional behaviour genetic study findings has prompted widespread discussion. Fundamental questions have been raised across the whole field of complex genetic traits as well as for behavioural traits. We consider explanations for recent findings and discuss what they mean for the field of developmental psychopathology.

Traditional family, twin and adoption studies have shown consistently that psychopathology and cognitive traits are familial and heritable. High heritability estimates have led to the conclusion that genetic factors make a substantial contribution to risk of many mental disorders and have fuelled the search for DNA variants associated with psychopathology and cognitive traits. Molecular genetic studies of behavioural traits and psychopathology involve direct assessment of variation across the whole genome using thousands of genetic markers (single nucleotide polymorphisms, SNPs). A new method of analysis assesses heritability that is attributable to the measured SNPs. Recent genome-wide association (GWA) analyses have provoked debate because they generate ‘SNP heritability’ estimates for behavioural traits that appear to be dramatically lower than those observed for other traits and when using traditional designs; although SNP heritability (*h*^2^) and traditional *h*^2^ are not the same. Findings from a recent twin-based molecular genetic study of cognitive and behavioural traits (Trzaskowski, Dale, & Plomin, 2013) were especially striking because it was possible to directly contrast traditional twin and SNP heritability estimates in the same sample, as well as compare estimates for behavioural, cognitive and physical measures. SNP heritability estimates were extremely low for behavioural traits, notably much lower than those for cognitive ability, height and weight in the same sample and very much lower than the twin-based heritabilities. The findings imply that the low estimates for the behavioural traits reflected something different about these traits rather than a problem with the experimental design.

The puzzle of the disparity between molecular- and traditional behaviour genetic study findings (Vinkhuyzen, Wray, Yang, Goddard, & Visscher, 2013; see also Wray et al. (2014) in this issue for explanations) has prompted widespread discussion. Fundamental questions have been raised across the whole field of complex genetic traits as well as for behavioural traits. Did traditional approaches, such as twin studies, in fact generate misleadingly high heritability estimates for psychopathology? Do genome-wide molecular genetic techniques have value in our field? It has been questioned whether genetic contributions at a molecular level are in fact trivial for psychopathology and behaviour.

Traditional behaviour genetic approaches statistically infer familial risk and heritability from observed patterns of phenotypic similarities between relatives who differ in their degree of biological relatedness (e.g. monozygotic and dizygotic twins, adoptive and biological parents’ of children). Whilst there are a number of important assumptions and limitations inherent in these designs (Tenesa & Haley, 2013), findings of consistently high *h*^2^ fuelled the impetus to identify genetic risk factors at a molecular level. Such investigations involve direct assessment of DNA variation (i.e., gene variants) and typically have exploited case**–**control comparisons or the correlation of DNA variation with trait variation in a population.

How do GWA studies assess genetic contributions and heritability? The design involves assessing hundreds of thousands of DNA markers that lie across the genome. Such studies typically assess only one class of DNA marker or variation, a common frequency single nucleotide (single nucleotide polymorphism, SNP). Variation in the whole genome is not measured; this is important as there are many other classes of DNA variation. Also, the measured SNPs should be viewed as risk markers because they are selected only to ‘tag’ or provide an indirect index of DNA variation that will include the truly causal SNPs. Whole genome molecular genetic designs have utilised three key approaches [see Wray et al. (2014) for a scientific account]. First, a key aim of these GWA studies (GWAS) was to identify single SNPs associated with disorders or traits. As adjustment for multiple testing is needed, and we have now learnt the effect size of any single common SNP is small, extremely large sample sizes (tens of thousands) are required to achieve genome-wide levels of statistical significance. A second, alternative approach has involved generating aggregate SNP risk scores known as genomic profile risk or polygenic scores that include individual SNPs which, may individually, fall below the stringent threshold for genome-wide significance. The third approach to examining molecular genetic contribution, relevant to the present discussion, uses all SNPs, not just those that are statistically significant or near significant. By assessing the degree of SNP similarity within cases and comparing this with SNP correlation within controls (or testing the relationship between SNP sharing and a quantitative phenotype), an indirect ‘heritability estimate’ or SNP *h*^2^ is generated. This analytic method is called GREML (genomic-relationship-matrix restricted maximum likelihood), sometimes referred to as GCTA after a software program in which this method is implemented (Vinkhuyzen et al., 2013; Wray et al., 2014).

Building from this brief synopsis of molecular genetic approaches to genetic risk estimation for psychopathology, the issues we are focusing on in the present contribution is why are SNP based *h*^2^ estimates for behavioural traits so much lower than those generated from traditional behaviour genetic designs and why does SNP *h*^2^ appear to be especially low for behavioural traits in comparison to other characteristics such as cognitive ability (Trzaskowski et al., 2013)? First, what is meant by heritability? It is a population attribute (see Tenesa and Haley (2013) for a detailed discussion on heritability), relies on a number of assumptions in traditional designs, and captures all types of genetic variation. Thus, traditional twin *h*^2^ includes contributions of common, rare and intermediate frequency alleles, DNA structural (e.g. small deletion) and sequence (nucleotide) variants, gene–gene interaction and gene–environment interaction effects. GCTA assesses additive effects of the measured common SNPs and is thus known as SNP heritability; additive SNP variance provides a more specific description. ‘SNP heritability’ is thus expected to be lower than twin heritability because they refer to different measures.

Would we be surprised, for example, if multiple measures of adversity in a twin study failed to explain all the environmental variance? They would be unlikely to explain all population-trait variance. We would realise that (a) our environmental measures likely provide an index rather than a direct assessment of each causal risk; (b) we have not captured all relevant environmental exposures and that (c) interactions between different environmental risks are also likely to be operating. That is also the case for GWAS where SNPs do not provide a direct assessment of every variety of causal genetic risk, and testing multiple interaction effects for every risk marker across the genome is not statistically feasible. Moreover; we would realise that the effects of environment and genes, whilst neatly partitioned for statistical purposes in behaviour genetic studies, are of course highly correlated and interdependent (Plomin, 2013; Rutter, 2012). These factors are important and cannot be easily ignored (Rutter, 2012). For example, there is clear evidence from simpler organisms such as yeast, worms and fruit fly that gene–gene interaction (epistasis; Mackay, 2014) and gene–environment interaction (e.g. Burns et al., 2012) make important contributions to variation in traits, such as epistasis in aggressive behaviour (Mackay, 2014) or adult exploratory activity in response to early nutritional adversity in *Drosophila melanogaster* (fruit fly). Gene-by-environment (G × E) interactions also occur across multiple biological levels. All such effects would be encompassed in a broad-sense heritability estimate from traditional studies but not by SNP heritability.

The study conducted by Trzaskowski et al. (2013) is, however, striking because GCTA picked up SNP additive variance for cognitive abilities but not for behavioural traits and the design allowed direct comparison of twin-inferred heritability and SNP additive variance. Does this mean, as suggested in some media quarters, that there is no evidence that genes or rather, common genetic risk variants (SNPs) influence psychopathology? That is not supported by broader research findings. Genome-wide significant individual SNP associations have now been detected in an adequately powered schizophrenia sample (Schizophrenia Working Group of the Psychiatric Genomics Consortium 2014). Composite SNP risk scores significantly distinguish cases from controls for ADHD, autism, schizophrenia, depression and bipolar disorder. Recent consortium analyses of these five psychiatric disorders (Cross Disorders Group, 2013) detected ‘SNP heritabilities’ ranging between 0.17 (lowest for autism spectrum disorder, ASD) to 0.28 (for ADHD).

Do these findings mean that common gene-variant contributions are restricted to those with a psychiatric diagnosis and are very much less prominent for behavioural traits? In general, statistical power is enhanced when selecting extremes. Also, those with a psychiatric diagnosis, especially those who are referred to clinics, likely score high on multiple trait measures (e.g. a child with ASD showing difficulties with social communication, restrictive repetitive behaviours, cognitive ability, language ability, behavioural problems and anxiety). Theoretically these factors could also contribute to higher enrichment of common genetic risk variants in those with a diagnosis. Nevertheless, there is evidence of SNP additive variance contributing to questionnaire-generated trait/behavioural measures from some studies. For example, a recent study showed significant ‘SNP heritability’ (0.18) for social communication/ASD traits (St Pourcain et al., 2013). Another found significant ‘SNP heritability’ (0.12–0.18) for subjective well-being (Rietveld et al., 2013). Finally, a recent study found that composite SNP risk scores derived from ADHD clinical cases (vs. controls) predicted ADHD trait measures in an independent general population (Martin, Hamshere, Stergiakouli, O'Donovan, & Thapar, 2014), a finding that is in keeping with the view that disorder, at least ADHD, lies at the extreme of normal trait variation.

Having established that SNP additive genetic variance has been found to contribute to some traits as well as psychiatric disorders, we can ask is it possible that different behavioural traits have different genetic architectures? That is, is SNP additive variance low for some traits, because other types of risk factors are more important? For example, it is possible that some traits are more strongly explained by rare genetic variants such as copy number variants (CNVs; Thapar & Cooper, 2013), gene-environment interaction and assortative mating (Trzaskowski et al., 2013) than SNP additive effects. That is certainly plausible but will need to be explicitly demonstrated rather than inferred through a failure to detect SNP additive variance.

Another issue to consider is whether measure and rater effects contribute to inflation of twin heritability estimates, differing patterns of SNP heritability for different traits as observed by Trzaskowski et al. (2013) as well as differences between behavioural traits and psychiatric disorder. Cognitive ability is assessed by performance on standard tests, and is an operationally robust variable. Behavioural traits (e.g. behaviour, hyperactivity, autistic traits) in twin studies are commonly assessed in the general population by questionnaire ratings. Molecular genetic studies of psychiatric disorders, such as ADHD, autism and schizophrenia, have typically involved clinic-referred patients who are assessed using interviewer-administered research diagnostic interviews. However, as already discussed SNP additive variance has been shown to contribute to questionnaire-assessed behavioural traits in some population cohorts at levels not much different than those identified for psychiatric disorder (Martin et al., 2014; Rietveld et al., 2013; St Pourcain et al., 2013). Thus, in our view it is premature to conclude that the genetic architecture of behavioural traits is necessarily complicated by rater and measure effects or is substantially different to that of psychiatric disorder. However, it is theoretically plausible that rater and measure effects contribute to inflated twin heritabilities for behavioural traits and that much larger general population samples are needed to detect SNP additive variance for some traits and measures.

In conclusion, as discussed in this issue (Wray et al., 2014) traditional heritability and ‘SNP heritability’ are different concepts and measures. We believe for the general reader, it would be preferable to think of SNP heritability as SNP additive variance to clarify this important distinction. In our view, however, the key issues for the field of child and adolescent psychiatry and developmental psychopathology are not to do with ‘missing’ or ‘still missing heritability’ quantities. Evidence from multiple studies clearly shows that common DNA variants in the population contribute to risk of psychopathology. Rather we believe it is important to consider how findings from current and future large scale genetic discovery studies can be used to address clinically and developmentally meaningful questions. For example, to examine how environmental risk and protective factors work with identified genetic risks and to identify potentially modifiable risk mediators and moderators underlying disorder and psychopathology trajectories. Finally, it will be critical to take forward promising genetic risk markers and examine these across a range of designs, in humans and other organisms, to test causality and identify causal risk mechanisms just as we would for any risk factor or set of risk factors.
